# Management of Chemotherapy-Induced Peripheral Neuropathy (CIPN) in Oncologic Patients—A New Promise? Preliminary Results

**DOI:** 10.3390/cancers17203321

**Published:** 2025-10-15

**Authors:** Ron Batash, Noam Asna, Sara HaJ Ali, Tatiana Charkovsky, Murad Asali, Sharon Pelles, Moshe Schaffer

**Affiliations:** 1 Orthopedic Oncology Unit, Tel Aviv University, Wolfson, Holon 5822012, Israel; 2 Oncology Department, The Hebrew University of Jerusalem, Sharei Zedek, Jerusalem 9103102, Israel; 3 Faculty of Medicine, Ben Gurion University, Beer Sheva 8499000, Israel; 4 Oncology Department, Barzilai Medical Center, Ashkelon 7830604, Israel; 5 Urology Department, Barzilai Medical Center, Ben Gurion University, Ashkelon 7830604, Israel; 6 Oncology Department, Tel Aviv University, Suarski Medical Center, Tel Aviv 6423906, Israel

**Keywords:** chemotherapy, CIPN, sarcoma, oncology, pain, peripheral neuropathy, ketamine, amitriptyline

## Abstract

**Simple Summary:**

Chemotherapy-Induced Peripheral Neuropathy (CIPN) is a well-known condition among oncologic patients. Up to 100% of oncologic patients including sarcoma patients treated with chemotherapy will develop CIPN. According to the literature, there is no effective treatment to offer to these patients. The aim of our study was to check the effects of a new combination of amitriptyline and ketamine given to these patients as a topical cream. We have followed 37 patients in our study and confirmed the results by using VAS scores to determine the alleviation of the pain and related symptoms.to address the condition. The new combination was proven effective and is considered useful for dealing with CIPN in oncologic patients.

**Abstract:**

Background: Chemotherapy-induced peripheral neuropathy (CIPN) is a common, often debilitating side effect of several chemotherapeutic agents, particularly taxanes and platinum compounds. Current management strategies remain limited, with few agents demonstrating consistent efficacy. Our aim was to evaluate the efficacy and safety of a novel topical cream containing 1% amitriptyline and 0.5% ketamine in alleviating CIPN symptoms in oncology patients. Methods: A prospective observational study was conducted at Barzilai Medical Center following the approval of the local ethics committee. Thirty-seven patients (30 females, 7 males; age 40–75) with CIPN following taxane or platinum-based chemotherapy were treated with a topical cream applied three times daily for 28 weeks. Pain intensity was assessed using the Visual Analog Scale (VAS). Inclusion and exclusion criteria were strictly applied to ensure a homogenous cohort. Results: Initial mean VAS was 7 (range 6–10), which decreased to a final mean VAS of 3 (range 1–7). Two patients withdrew from the study, and 27 out of 35 evaluable patients (77.14%) reported significant symptoms relief. A statistically significant reduction in pain scores (*p* = 0.00005) was seen in our study. Only mild, transient erythema was reported as a side effect. Conclusions: This is the first clinical evaluation of a topical combination with new concentrations of amitriptyline and ketamine for CIPN. The results suggest this formulation offers a safe and effective option for symptomatic relief. Larger randomized controlled trials and exploration of transdermal delivery systems are warranted to validate these findings and optimize treatment outcomes.

## 1. Introduction

Peripheral neuropathy is defined as damage to the peripheral nervous system, which may result from trauma, metabolic disorders, or medical treatments. Common causes include Diabetes Mellitus, Autoimmune diseases, and Chemotherapy exposure. Neuropathy manifests with a wide spectrum of motor and sensory symptoms, including limb weakness, sensory loss, paresthesia, burning pain, cramps, gait imbalance, and impaired coordination [1,2,3,4].

CIPN is a dose-limiting and debilitating complication of cancer treatment, characterized by partially reversible or long-lasting effects [4,5]. The incidence varies between 20% and 100%, with early manifestation during therapy in up to 40% of patients. Severe neuropathy develops in 3–7% of monotherapy cases and up to 38% with combined regimens [6]. Patients with pre-existing neuropathy (e.g., diabetes mellitus, hereditary neuropathy, connective tissue disease) are particularly vulnerable, sometimes developing symptoms after a single chemotherapy cycle [7].

Among the chemotherapeutic classes, platinum-based agents such as oxaliplatin, cisplatin, and carboplatin are most frequently associated with neuropathy. These drugs are widely used in the treatment of colorectal, anal, and pancreatic carcinomas, as well as lymphoma and osteosarcoma. Neuropathy is typically sensory-predominant, characterized by paresthesia, cold sensitivity, and numbness. Symptoms are often persistent and represent a major barrier to treatment adherence. (A summary of the regimens associated with peripheral neuropathy is provided in Table 1).

Taxanes, including paclitaxel and docetaxel, are commonly used for breast, ovarian, uterine, and prostate cancers. Neuropathy associated with taxanes is dose-dependent and primarily sensory in nature. Although symptoms may improve after discontinuation, they are often long-lasting. Notably, paclitaxel carries a higher risk of neurotoxicity compared to docetaxel, and neuropathy is a frequent cause of treatment reduction or interruption.

Vinca alkaloids, particularly vincristine and vinblastine, play a central role in the treatment of hematologic malignancies, such as lymphoma, leukemia, and sarcoma. These agents are associated with severe neurotoxicity, which can involve sensory, motor, and autonomic fibers. Neuropathy may occur even at relatively low doses, and its severity often limits further chemotherapy.

Another important class includes proteasome inhibitors, such as bortezomib, which are integral to the treatment of multiple myeloma. Bortezomib-induced neuropathy is usually painful and sensory-predominant. Subcutaneous administration has been shown to reduce the risk compared to intravenous delivery, although neurotoxicity remains a significant clinical issue.

Immunomodulatory drugs, particularly thalidomide and lenalidomide, are also linked to peripheral neuropathy in multiple myeloma patients. Thalidomide has a well-documented association with chronic, often irreversible sensory neuropathy, while lenalidomide carries a lower but still notable risk of neuropathic complications.

In sarcoma-specific regimens, the most commonly used agents include doxorubicin as first-line therapy and ifosfamide in combination. In advanced or metastatic settings, additional agents such as docetaxel, gemcitabine, and dacarbazine are employed [8,9]. Neuropathy in this population is frequently linked to high cumulative doses of doxorubicin and ifosfamide [10]. According to Earl et al., nearly 50% of patients with soft tissue and bone sarcomas developed CIPN after a median of 8 months of treatment (range: 1–54 months) [11].

The pathophysiology of CIPN still remains vague at the level of the nervous system and neural structures. Gordon-Williams et al. described different potential mechanisms responsible for the neuropathy of such patients, e.g., neuronal cytoskeleton changes, damage to neuron/DRG in the mitochondria, impairment of ion channels function, glial cell activation, and microtubules disruption, which result in a reduced nerve conduction velocity and action potential amplitude following oxaliplatin therapy, together with a neuronal atrophy [12].

The management of chemotherapy-induced peripheral neuropathy remains largely symptomatic, as no curative therapy is currently available. Several treatment modalities have been explored, ranging from pharmacological approaches to non-pharmacological and device-based interventions. (A short summary of the Treatment options for chemotherapy-induced peripheral neuropathy (CIPN) can be found in Table 2). 

Pharmacological therapies include systemic agents such as tricyclic antidepressants (e.g., amitriptyline), anticonvulsants, ketamine, cannabinoids, and opioids. These drugs have demonstrated some efficacy in reducing neuropathic pain and paresthesia; however, they remain predominantly palliative and are associated with significant limitations, including sedation, dependency potential, and systemic adverse effects [13]. Topical formulations—such as capsaicin, lidocaine, and opioid-based gels or patches—have been used in refractory cases with variable success. While they may provide localized symptom relief, their durability is limited, and local irritation or other adverse reactions must be considered [14].

Non-pharmacological interventions such as physical therapy, structured exercise programs, and acupuncture have shown potential benefits in improving pain relief and functional outcomes. Nonetheless, evidence from randomized controlled trials is heterogeneous, and guidelines have not reached a consensus regarding their efficacy [15]. Similarly, neuromodulation techniques, including transcutaneous electrical nerve stimulation (TENS) and neurofeedback, are being investigated, but current data are limited, and protocols are not standardized [15].

Photobiomodulation (PBM) therapy—also referred to as low-level laser therapy (LLLT)—has gained increasing attention. Several randomized controlled trials using 630/850 nm wavelengths demonstrated significant improvements in sensory symptoms, gait, and balance, with patient-reported benefits persisting for up to 12 weeks after treatment cessation [16,17,18,19]. Preliminary data suggest that PBM may also have a role in prevention, when administered concurrently with chemotherapy, by reducing the severity of neuropathic symptoms and improving quality of life [20]. Despite its favorable safety profile and low incidence of adverse effects, the evidence remains insufficient due to heterogeneous study designs, small sample sizes, and variability in treatment parameters. Consequently, PBM has not yet been adopted into ASCO clinical guidelines for the management of CIPN [21].

Overall, while multiple therapeutic strategies are available, their effectiveness is inconsistent, and none have emerged as a standard of care. This highlights a significant unmet clinical need, particularly for therapies that are both effective and well-tolerated.

Based on experience and evidence suggesting that amitriptyline and ketamine have independently shown a reduction in CIPN symptoms, mainly pain, we have investigated a novel formulation of the two agents, aiming to enhance the therapeutic effects of these components.

The purpose of this preliminary study was to assess the efficacy and therapeutic response of a new combination of Amitriptyline (1 gram (g)) and Ketamine (0.5 g) in oncologic patients suffering from Chemotherapy-Induced Peripheral Neuropathy.

## 2. Materials and Methods

After following the approval of the local ethics committee to a limited number of participants due to the regulations, 37 consecutive patients suffering from Chemotherapy-Induced Peripheral Neuropathy were included in a preliminary observational study.

A combination of Amitriptyline and Ketamine was composed after consultation with our medical center pharmacologist and given to subjects with chemotherapy-induced peripheral neuropathy symptoms. All patients were evaluated according to Visual Analog Scale (VAS) on several occasions during their follow-up visits at the oncologic department of Barzilai Medical Center, Ashkelon. The efficacy of the compound was determined according to the reports of the patients during a period of 28 weeks, with correlation to the VAS score reported by the patients during their outpatient clinic visits.

A tube of 100 g of DAC-based cream containing 1 g of Amitriptyline and 0.5 g of Ketamine was prepared and given to the patients to be applied to the designated affected skin. The compound was administered in the form of cream to be put on with a daily routine of three times a day, a thin layer, on the area affected by the neuropathy. After application, the patients were ordered to cover the creamed skin for about an hour to prevent light sensitivity.

Inclusion criteria for the study were as follows: adult oncology patients aged 18 years and older who were receiving chemotherapy—either adjuvant or neoadjuvant—with no more than three cycles completed, including regimens containing taxanes or platinum-based agents. Eligible participants must have completed chemotherapy no more than 2 months prior to study enrollment, reported a pain score of 6 or higher on the Visual Analogue Scale (VAS), and be willing to initiate or adjust analgesic medications, excluding NSAIDs. Additionally, all participants were required to be capable of providing written informed consent.

Exclusion criteria included patients with a poor prognosis—defined as less than 6 months of overall survival; those with concomitant medical conditions such as renal insufficiency or diabetes mellitus; individuals with neurological disorders or peripheral nerve damage caused by other illnesses or medications; and patients receiving antiepileptic or antidepressant drugs with unstable dosing prior to enrollment. Additional exclusions were ongoing or planned treatment during the study with amitriptyline, ketamine, or any cannabinoid preparations; participation in other investigational treatments within 1 month prior to enrollment; low anticipated compliance; an ECOG performance status of 4 or higher; known allergies to the substances under investigation; significant dermatological conditions; and pregnancy.

The statistical analysis was performed using SPSS v.29 software. A Wilcoxon signed-rank test was chosen to demonstrate the effectiveness of the treatment in accordance with VAS changes.

## 3. Results

Thirty-seven consecutive patients (7 males, and 30 females), aged between 40–75 years old, suffering from CIPN based on Taxol or Platinum, were included in the study. Two patients were lost to follow-up. All patients received a topical cream containing 1 g of Amitriptyline and 0.5 g of Ketamine. The patients were recruited for the treatment at the end of their chemotherapy or up to 2 months after starting their treatment.

All patients were assessed by VAS, a unidimensional measurement for pain intensity, which has been widely used in variety of adult patients, including oncology, rheumatology, and orthopedics. The result of VAS = 0 was regarded as pain-free, while VAS = 10 was considered to be the worst pain. A minimally clinically meaningful effect was defined as a reduction of 3 points on the VAS scale, as reported by the patients following the application of the drug. To our knowledge, no other pain medication drug was taken by our patients along with our experimental compound.

**Figure 1:**
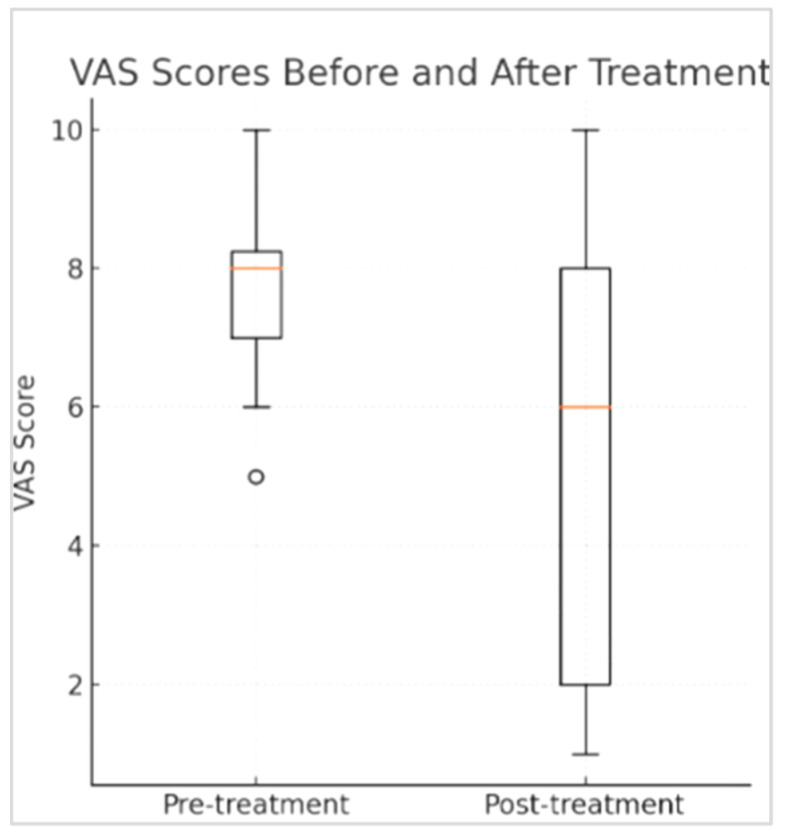
Boxplot demonstration of median VAS scores before and after application of the cream.

In our study, the minimal initial VAS score was 6. The Mean initial VAS score was 7.94 (range 6 to 10), median = 8, prior to the cream administration, and the Mean final VAS score was 5.5 (range 1 to 7), median = 6. The effect size was Z -4.89, r = 0.82. Two patients left the study; one due to progression of her base illness, while the other failed to comply with the treatment protocol. Of our 35 recruitment patients, 10 patients did not respond to the treatment (28.58%), while 25 patients (71.42%) responded to the treatment. Most of the patients responded well to the treatment, with a reduction of VAS scores > 3 points during the first 2 weeks of the study. However, we have noticed that after the drug was stopped due to completion of the chemotherapy treatment, a partial relapse was seen in their CIPN symptoms. Therefore, we must repeat the study on a larger scale. See Figure 1.

The Side effects reported were only mild, transient, self-limited skin Erythema.

**Figure 2:**
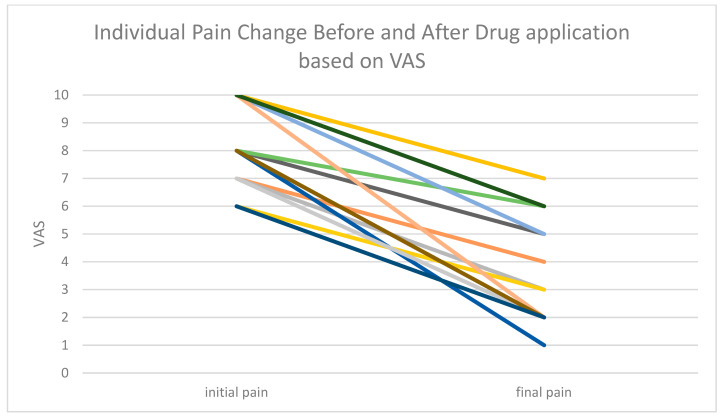
Effects of the Drug administration on VAS reported by patients (initial and final pain) With statistical significance *p* = 0.0000878. Each colored line represents a different patient with initial and final VAS measurements.

A Wilcoxon signed-rank test was performed to evaluate the correlation of the drug and the reduction in VAS, and statistical significance *p* = 0.0000878 was demonstrated. See Figure 2.

## 4. Discussion

Chemotherapy-Induced Peripheral Neuropathy is considered a complex and challenging condition for treatment. According to the American Society of Clinical Oncology (ASCO), there is no agreement on the ideal method of prevention and treatment of CIPN patients [21].

According to ASCO guidelines, Duloxetine is the only pharmacologic agent recommended for chemotherapy-induced peripheral neuropathy (CIPN) pain management based on moderate evidence of efficacy. Over the years, several studies regarding novel pharmacologic agents, including pregabalin, gabapentin, tricyclic antidepressants, and venlafaxine, were conducted, but due to weak or inconsistent evidence supporting their use, they are not formally recommended by ASCO guidelines [21,22,23].

The combined approach of non-pharmacologic treatment for CIPN, including the use of low-level laser therapy (photobiomodulation, PBM) in conjunction with exercise or physical therapy, has been examined, but current evidence does not demonstrate a clear, significant benefit from combining these modalities over PBM or exercise alone.

Argenta et al. conducted a randomized, sham-controlled trial that directly evaluated PBM with and without physiotherapy in patients with established CIPN. Both PBM alone and PBM plus physiotherapy patients showed a significant and clinically meaningful reduction in neuropathic symptoms, as measured by the modified Total Neuropathy Score, with improvements sustained up to 16 weeks. However, when physiotherapy was added to the protocol, the patients did not report further enhanced outcomes compared to PBM alone, which indicates no clear synergistic effect between these interventions in this setting [19].

Separately, PBM has been shown to improve balance, sensory symptoms, and gait speed in CIPN patients, while physical therapy and neuromuscular training independently reduce CIPN severity, improve physical function, and enhance quality of life [18,24]. Streckmann et al. [25] reported that some exercise interventions, including sensorimotor and whole-body vibration training, decrease the onset of CIPN. Yet, the current clinical evidence does not show superiority for combined PBM and exercise/physical therapy over either intervention alone for CIPN, though both are safe and effective modalities. Multimodal approaches remain reasonable, especially when tailored to patient needs, but further research is needed to clarify optimal integration [25].

The underlying mechanisms of chemotherapy-induced peripheral neuropathy (CIPN) differ from other types of neuropathies, including diabetes-induced neuropathy and autoimmune neuropathy; however, there are similar described pathologic pathways, such as neuroinflammation or axonal degeneration.

Chen et al. Described the direct neurotoxic effects of chemotherapeutic agents on peripheral neurons, especially dorsal root ganglia and sensory axons [26]. Several main mechanisms responsible for CIPN were studied and mentioned in the literature, including mitochondrial dysfunction, disruption of microtubule function, DNA damage induction, alteration of ion channel expression, oxidative stress, DNA damage, and neuroinflammatory responses, which involve dorsal root ganglia and immune cells. Moreover, the type of chemotherapy chosen for the treatment of oncologic patients affects the different molecular targets and described pathways, which results in different patterns of injury. Yet, the main insult is a neuropathy or sensory-predominant axonopathy [27,28,29,30,31,32].

According to recent review studies, diabetic neuropathy is driven by chronic metabolic changes, including the activation of the polyol pathway due to a hyperglycaemic state, oxidative stress, advanced glycation end-product formation, microvascular insufficiency, and low-grade inflammatory reaction. These mechanisms result in a progressive nerve ischemia, demyelination, and axonal loss; however, the initiating factors are metabolic rather than toxic [33,34,35].

Autoimmune neuropathies, such as Guillain-Barré syndrome, are characterized by axonal injury or immune-mediated demyelination, which are triggered by autoantibody production or molecular mimicry against nerve elements. The described pathogenesis involves infiltration of macrophages, activation of complement, and nerve damage due to antibodies, as opposed to metabolic injury seen in diabetic neuropathy or direct toxicity seen in CIPN patients [36,37,38].

In our study, we have introduced a groundbreaking solution using a combination of 1 g of Amitriptyline and 0.5 g of ketamine as a topical cream for CIPN patients. To our knowledge, this is the first clinical trial with the new suggested composition of this compound.

The use of topical amitriptyline and ketamine targets nociception, whereas in diabetic patients, this combination has demonstrated an analgesic effect. Barton et al. evaluated a topical compound containing 40 mg of amitriptyline and 20 mg of ketamine in 1.3 g of the solution versus a placebo and demonstrated slight improvement in sensory neuropathy and motor subscale improvement. However, when a combination of topical 2% ketamine and 4% amitriptyline cream was used, the patients failed to demonstrate improvement in CIPN. Based on the experiment of Barton et al. it could be explained that the positive effect in those patients depends on the concentration of the two main substances and frequency of use [39].

According to the literature, there have been several trials to administer the described materials, e.g., vaporized cannabinoid, topical solutions, Intravenous, yet none have proven to be effective for the condition [40].

Tetrahydrocannabinol (THC) is a phytocannabinoid that was found to be effective in paclitaxel-, oxaliplatin-, and vincristine-induced peripheral neuropathy. King et al. Showed in vitro studies that THC or CBD alone attenuates mechanical allodynia associated with paclitaxel-induced peripheral neuropathy; however, when THC is combined with cannabidiol (CBD), a synergistic effect leads to reduced neuropathic pain [41]. THC’s efficacy is considered agent-specific: it is effective in vincristine- and paclitaxel-induced neuropathy, but less effective in case of oxaliplatin-induced neuropathy. The mechanism of action of THC is mainly via CB1 and CB2 cannabinoid receptors, which modulate pain signaling and neuroinflammation due to a mixed agonist effect. According to Barnes et al., when Cannabinoid agonists, e.g., AM1241, ACEA, and CP55, are administered, tolerance and gender-specific responses were noticed, with females developing tolerance more rapidly than males [42]. On the other hand, CB2-selective agonists do not show such tolerance effect, which can be considered as an advantage for targeting CB2 in CIPN [43].

Despite new evidence from case series and narrative reviews suggesting that THC may offer symptomatic relief in CIPN patients, high-quality clinical evidence remains limited. To date, randomized controlled trials evaluating the efficacy and safety of THC in this context are lacking.

Preclinical studies have demonstrated the potential of THC to alleviate CIPN-related symptoms, supporting its biological plausibility as a therapeutic agent. Nonetheless, its integration into current clinical practice guidelines has not been established. While the safety profile of THC is generally favorable, its psychoactive properties and the potential for tolerance development necessitate cautious consideration in clinical decision-making [42,44].

The use of THC together with CBD and separately as a topical cream is absorbed into the skin based on the interaction with the epidermis and dermis layers. On one hand, the absorbed material is effective locally for pain relief and inflammation; on the other hand, the active compound does not penetrate deeply enough to reach the bloodstream in significant amounts. The CBD and THC interact with the cannabinoid receptors. Hence, systemic absorption is limited.

There are several known factors that affect the absorption:

1. the particle size of the applied material: there is an inverse relation between the size and penetration. The smaller the particle, the better the penetration via the skin.
2. The carrier ingredients: There are several materials that are known for their capability for enhancing the penetration, e.g., ethanol.
3. The formulation of the cream: as depicted above, there have been several compositions in the past with different concentrations of the same compounds. The Level of absorption was different every time up to complete failure to penetrate the skin and reach the desired goals.
4. The integrity of the skin prior to the treatment can affect the absorption; the more damage to the skin, the higher the absorption.

In the literature, there have been reports of transdermal applications in the form of patches. Chu et al. demonstrated that the transdermal flux of CBD was approximately 13.25 ng/cm^2^/h and cumulative drug permeation of 610.96 ± 88.92 ng/cm^2^ over 24 h. Shankar et al. have proven that the transdermal delivery of amitriptyline resulted in significant skin concentrations with negligible plasma levels. Such products are designed to deliver the CBD compounds to the bloodstream and allow systemic absorption.

Ozone treatment, in the context of CIPN, was introduced by Clavo et al. In that study, ozone was administered via rectal insufflation to CIPN patients, where improvements in numbness, pain, and tingling were reported by the participants, which suggests a possible therapeutic option. However, Due to the low number of patients in the groups’ preliminary results, the quality of evidence is considered insufficient to be included in the ASCO guidelines [45,46].

Duloxetine, a selective serotonin and norepinephrine reuptake inhibitor (SNRI), was found in several studies to be effective in the treatment of CIPN patients and even considered as the only agent with moderate evidence and support by the guidelines [47]; however, a recent study demonstrated the low outcomes of duloxetine when compared to placebo in colorectal patients developing or preventing CIPN, hence the role of duloxetine still remains debatable according to ASCO [48].

It is possible that the solution to the symptoms of neuropathy depends on the combination of ketamine and amitriptyline with, for example, ozone, THC, or CBD and other cannabinoids, both in combination with ointment, use of a patch, and sometimes administration of some of the atypical drugs orally and intravenously.

In this study, we demonstrated the reduction of pain in CIPN patients using a novel topical combination of 1 g of ketamine and 0.5 g of amitriptyline. As a preliminary investigation, the study has several limitations. It was a non-randomized, single-arm trial conducted under the constraints of our local ethics committee, which precluded the inclusion of a control group or comparison with alternative treatment modalities, e.g., cannabinoids, physical therapy, etc. These limitations restrict the strength of our clinical recommendations.

Our findings provide a rationale for future studies to further evaluate this therapeutic approach, including its potential application in other neuropathic pain-associated diseases, as mentioned earlier. Larger, multi-arm studies with longer follow-up periods are warranted to confirm efficacy. Moreover, other methods of administration, such as transdermal patches, should be explored to increase absorption, drug release, and potentially reduce the frequency of daily applications.

In our study, we have managed to demonstrate the effectiveness of topical compound with the composition of 1% Amitriptyline, 0.5% Ketamine. This composition has managed to significantly reduce the main symptoms of pain in our oncologic patients suffering from CIPN.

## 5. Conclusions

In summary, while all CIPN, Diabetic, and autoimmune neuropathies share final common pathways of nerve injury and neuroinflammation, the initiating mechanisms and primary pathogenic processes are distinct for CIPN, diabetic neuropathy, and autoimmune neuropathies. Considering the findings given, our preliminary results provide a rationale for further studies to establish effective therapeutic modalities for CIPN patients.

## Figures and Tables

**Table 1:** Chemotherapy regimens associated with peripheral neuropathy.

Chemotherapy Drug	Main Indication	Neuropathy Symptoms	Clinical Impact
**Platinum agents** (Oxaliplatin, Cisplatin, Carboplatin)	Colorectal, Pancreatic, and anal cancer, Uterus, Ovarian, lymphoma, osteosarcoma	Sensory neuropathy, parasthesia, cold sensitivity, numbness	May require discontinuation or dose reduction
**Taxanes** (Paclitaxel, Docetaxel)	Breast, Ovarian, Uterine, and Prostate cancers	Dose-dependent sensory neuropathy, higher incidence in paclitaxel	Long-lasting; major cause of treatment modification
**Vinca Alkaloids** (Vincristine, Vinblastine)	Lymphoma, Leukemia, and Sarcoma	Severe sensory and motor neuropathy	Neurotoxicity even at low doses. Can limit therapy
**Proteasome inhibitors** (Bortezomib)	Multiple myeloma	Painful sensory neuropathy, reduced with subcutan. administration	Can compromise treatment adherence and affect quality of life
**Immunomodulators** (Thalidomide, Lenalidomide)	Multiple Myeloma	Thalidomide poses high risk of chronic sensory neuropathy. Lower risk in case of Lenalidomide	Limit long-term use. High impact on daily function
**Anthracyclines and alakalying agents** (Doxorubicin)	Soft tissue sarcoma, bone sarcoma	Risk increases with high cumulative doses. Delayed neuropathy is possible	Nearly 50% develop CIPN within 8 months
**Other agents** (Docetaxal, Gemcitabine, Dacarbazine)	Advanced/metastatic sarcoma	Neuropathy varies by regimen	Cumulative burden when combined with other drugs

**Table 2:** Treatment options for chemotherapy-induced peripheral neuropathy (CIPN).

Treatment	Agents	Efficacy	Adverse Effects/Limitations
Pharmacological	Amiytriptyline, anticonvulsants, Ketamine, Cannabinoids, Opioids	Some pain and paresthesia relief, mostly symptomatic, not curative	Sedeation, dependency, systematic side effects
Topical	Capsaicin, Lidocaine, Opoid gels	Useful in refractory cases, can improve localized pain	Local irritation, limited durability
Non-pharmacological	Physical therapy, Exercise, Acupuncture	Improve function and pain relief in some RCT	Evidence heterogeneous, inconsistent recommendations
Neuromodulation	TENS, neurofeedback techniques	Under investigation	Limited data.
Photobiomodulation/low level laser	630/850 nm light	RCTs show improved sensory sensation, gait, and balance, with durability up to 12 weeks	Heterogeneous protocols, small cohorts; no consensus or recommended guidelines.
Preventive strategies	PBM applied during chemotherapy.	May reduce severity of symptoms and improve quality of life.	Preliminary data only; not included in ASCO guidelines.

## Data Availability

The raw data supporting the conclusions of this article will be made available by the authors on request.
